# Supplementary material for: Survey on the association between Toxoplasma gondii infection and violent behavior in inmates
Source: PLoS One. 2023 Apr 7;18(4):e0284202. doi: 10.1371/journal.pone.0284202 (PMC10081799; doi:10.1371/journal.pone.0284202)
Supplement: Attachment — Submitted filename: Response to reviewers Tg-Violence-3-PO.docx [file pone.0284202.s002.docx]

**RESPONSE TO THE REVIEWERS’ COMMENTS**

Journal Requirements:

Please review your reference list to ensure that it is complete and correct. If you have cited papers that have been retracted, please include the rationale for doing so in the manuscript text, or remove these references and replace them with relevant current references. Any changes to the reference list should be mentioned in the rebuttal letter that accompanies your revised manuscript. If you need to cite a retracted article, indicate the article’s retracted status in the References list and also include a citation and full reference for the retraction notice.

**References were reviewed and it is complete and correct. No retracted papers were found.**

**Thank you for your valuable comments for improving our manuscript.**

Reviewer #1: The changes in odds ratio values are shown in Table 2. However, the calculations and results of some ORs are not clear. For example, in the first row, the predicted value (OR = 0.5*105.5/6.35*17.5=0.4673) is 0.4637, but the reported value does not match it. Please explain to me how you can calculate this odds ratio.

**OR with zero values were recalculated and corrected.**

**Thank you for your valuable comments for improving our manuscript.**
